# Abnormal EEG microstates in Alzheimer’s disease: predictors of β-amyloid deposition degree and disease classification

**DOI:** 10.1007/s11357-024-01181-5

**Published:** 2024-05-10

**Authors:** Yibing Yan, Manman Gao, Zhi Geng, Yue Wu, Guixian Xiao, Lu Wang, Xuerui Pang, Chaoyi Yang, Shanshan Zhou, Hongru Li, Panpan Hu, Xingqi Wu, Kai Wang

**Affiliations:** 1 Department of Neurology, The First Affiliated Hospital of Anhui Medical University, the School of Mental Health and Psychological Sciences, Anhui Medical University, 218 Jixi Road, Hefei, 230032 Anhui China; 2 Anhui Province Key Laboratory of Cognition and Neuropsychiatric Disorders, Hefei, 230022 China; 3 Institute of Artificial Intelligence, Hefei Comprehensive National Science Center, Hefei, 230088 China; 4 Collaborative Innovation Center of Neuropsychiatric Disorders and Mental Health, Hefei, 230022 China; 5 Anhui Provincial Institute of Translational Medicine, Anhui Medical University, Hefei, 230032 China; 6 Department of Sleep Psychology, The Second Hospital of Anhui Medical University, Anhui Medical University, Hefei, 230032 China

**Keywords:** Alzheimer’s disease, EEG microstates, Cognitive function, CSF biomarkers

## Abstract

Electroencephalography (EEG) microstates are used to study cognitive processes and brain disease-related changes. However, dysfunctional patterns of microstate dynamics in Alzheimer's disease (AD) remain uncertain. To investigate microstate changes in AD using EEG and assess their association with cognitive function and pathological changes in cerebrospinal fluid (CSF). We enrolled 56 patients with AD and 38 age- and sex-matched healthy controls (HC). All participants underwent various neuropsychological assessments and resting-state EEG recordings. Patients with AD also underwent CSF examinations to assess biomarkers related to the disease. Stepwise regression was used to analyze the relationship between changes in microstate patterns and CSF biomarkers. Receiver operating characteristics analysis was used to assess the potential of these microstate patterns as diagnostic predictors for AD. Compared with HC, patients with AD exhibited longer durations of microstates C and D, along with a decreased occurrence of microstate B. These microstate pattern changes were associated with Stroop Color Word Test and Activities of Daily Living scale scores (all *P* < 0.05). Mean duration, occurrences of microstate B, and mean occurrence were correlated with CSF Aβ _1–42_ levels, while duration of microstate C was correlated with CSF Aβ _1–40_ levels in AD (all *P* < 0.05). EEG microstates are used to predict AD classification with moderate accuracy. Changes in EEG microstate patterns in patients with AD correlate with cognition and disease severity, relate to Aβ deposition, and may be useful predictors for disease classification.

**Supplementary Information:**

The online version contains supplementary material available at 10.1007/s11357-024-01181-5.

## Introduction

Alzheimer’s disease (AD), a pervasive neurodegenerative disorder, undermines various cognitive functions and affects roughly 40 million individuals globally [1]. With dementia predominated by AD, projections indicate a tripling in prevalence by 2050, which could escalate if adopting a biological over a clinical definition [2]. Presently, AD diagnosis leans on biomarkers like PET neuroimaging or CSF analysis—methods that are invasive or expensive, thus impractical for mass screening [3].

The aggregation of amyloid beta (Aβ) peptides in AD primarily affects specific brain functional networks that collaborate to perform tasks. Impaired brain network connectivity is associated with the clinical severity and cognitive function of AD [4–6]. It has been proposed that these disrupted connectivity patterns within large-scale networks manifest the diverse clinical symptoms and cognitive impairments across dementia variants [7–9]. While fMRI remains the standard for studying functional brain networks, its temporal resolution falls short in capturing the fleetingly dynamic nature of these networks, crucial during resting states [10]. Conversely, EEG microstates may offer a promising parallel, potentially mirroring the resting-state networks and the instantaneous activity of spontaneous BOLD recognized by fMRI, due to their superior temporal resolution [10, 11]. Hence, EEG stands out as an advantageous tool for screening AD patients due to its cost-effectiveness, non-invasive nature, and high temporal resolution, potentially serving as a supplementary method in detecting dementia biomarkers [12].

Our study delves into EEG microstate analysis, a cutting-edge technique that segments the EEG signal into a sequence of short-lived, stable brain topographies microstate potentially reflecting the electrophysiological footprint of different cognitive processes [13–15]. These EEG microstates are considered the “atoms” of thought, the smallest building blocks of mental activity, and are linked to the brain’s spontaneous activity during rest [15]. Our focus on microstates stems from their proven synchrony with the brain's default mode network, as detected by fMRI, suggesting that they may provide a window into the real-time dynamics of neural connectivity [16]. Furthermore, alterations in these microstates have been associated with various neurological disorders, including AD, where they may reflect disruptions in neural communication and integration [17–19]. By examining the nuances of these microstates, we aim to unearth subtle biomarkers of AD, contributing to a more nuanced understanding of the disease and enhancing the potential for early detection.

Although the altered pattern of EEG microstates in patients with AD and its spatiotemporal correspondence with the intrinsic network of resting-state fMRI has been investigated [12, 20–23], the linkage to biomarkers such as Aβ and tau proteins is less examined [24, 25]. The impact of these biomarkers on EEG microstate configurations and the resulting cognitive decline has not been fully explored, leaving a gap in understanding the pathological underpinnings and their cognitive implications in AD.

Based on the above, we propose the following hypotheses: (i) a reduction in dynamic brain activity, evidenced by microstate dynamics, is indicative of AD and correlates with clinical symptoms and cognitive impairment. (ii) Disruptions in resting-state EEG microstate dynamics in AD patients might be associated with the accumulation of biomarkers like Aβ and tau proteins, which could serve as predictors for the extent of abnormal protein accumulation. We posit that resting-state EEG microstate metrics could serve as ancillary indicators for AD biomarkers, potentially offering novel insights into the disease's pathophysiology and aiding early diagnosis.

## Methods

### Participants

We recruited 56 patients with AD from the Memory Disorders Clinic of the First Affiliated Hospital of Anhui Medical University and 38 sex- and age-matched healthy controls (HC) from the surrounding community. All patients with AD were diagnosed with probable AD dementia according to the NIA-AA criteria [26]. The AD group consisted of individuals aged between 50 and 80 years old who were right-handed, and Clinical Dementia Rating(CDR) is between 0.5 and 2. The control group comprised healthy elderly individuals who closely matched the age, sex, and educational level of the AD group. All participants underwent comprehensive medical and neuropsychological assessments, as well as resting-state EEG. Patients in the AD group underwent CSF examinations to assess levels of Aβ_1-42_, Aβ_1-40_, phosphorylated-tau, total-tau, and neurofilament light chain protein. None of the participants exhibited neurological or imaging abnormalities unrelated to AD, such as normal pressure hydrocephalus or widespread microinfarction. Furthermore, none had a history of secondary dementias, serious mental illness, alcohol abuse, or drug abuse. This study was approved by the Ethics Committee of Anhui Medical University, and all participants provided written informed consent. All procedures were performed by the Declaration of Helsinki in its current form.

### Neuropsychological assessments

All participants underwent exhaustive neuropsychological assessments by experienced neuropsychological examiners. The following neuropsychological tests were used to comprehensively evaluate cognitive function and clinical symptoms: Mini-Mental State Examination (MMSE), Montreal Cognitive Assessment (MoCA), Lawton-Brody Activities of Daily Living Scale (ADL), CDR, Global Deterioration Scale (GDS), Hamilton Anxiety Scale (HAMA), and Hamilton Depression Scale (HAMD). To assess individual cognitive domains, several tests were utilized. The Chinese Auditory Verbal Learning Test (CAVLT-Immediate, delayed, and recall, [CAVLT-I/D/R]), digital span test (forward/backward [DST-F/B]), Stroop color-word tests (SCWT-dot, words, and colored words [SCWT-D/W/CW]), and A verbal fluency test (letter/sematic [VFT-L/S]).

### EEG acquisition and preprocessing

EEG recordings were performed in a soundproof, dimly lit room equipped with a comfortable chair. All participants were instructed to wash their hair and ensure their scalp remained dry. A scalp elastic cap with 64 electrodes was applied following the International 10–20 system for EEG data recording (Neuro Scan, Sterling, VA, USA). During the recording, participants were instructed to close their eyes and sit quietly for 10 min while remaining awake to prevent drowsiness or fatigue. For resting-state EEG acquisition, the electrode positioned between FPz and Fz served as the ground, while EEG activity was recorded using the left mastoid electrode as an online reference [27]. A horizontal eye electrogram was placed 1 cm outside the bilateral eye corners. Vertical electrooculograms were positioned above the midpoint of the left eyebrow and 1 cm below the lower eyelid. The scalp resistance of all electrodes was maintained below 5 kΩ. The collected EEG data were displayed on an amplifier connected to another computer. The amplifier was set to 0.1–100 Hz bandpass filtering and 1000 Hz continuous sampling.

The resting-state EEG data were analyzed using the MATLAB software (R2013b, The MathWorks Inc., Natick, MA, USA) and the EEGLAB (R13_0_0b) toolbox [28]. Initially, bandpass filtering and dip filtering were applied to the raw data from 60 channels, excluding bilateral mastoid and EOG twin electrodes, with frequencies set at 0.1 to 40 Hz and 48 to 52 Hz, respectively. After downsampling to 500 Hz, the clean data were divided into non-overlapping 2-s segments. Channel data were scrutinized for integrity, with faulty channels being identified and interpolated based on rigorously defined criteria: abnormal amplitude excursions beyond ± 100 µV, sustained flat-line activity, and signal quality that deviated markedly from adjacent channels. Segments with noise—characterized by amplitudes surpassing ± 100 µV, transient spikes, or rapid frequency fluctuations uncharacteristic of neural origins—were excised from the dataset. On average, we found that approximately 3 channels per dataset were identified as noisy and subsequently corrected through interpolation. This figure is based on the aggregate data from all subjects. Independent component analysis (ICA) was subsequently deployed to isolate and exclude components typifying eye movements, head movements, electrode discontinuities, and muscle artifacts. Rejection of components adhered to predetermined thresholds for their spatial footprint, temporal dynamics, and frequency signatures that were distinctly non-neural—for instance, those predominantly active within muscle artifact frequency bands or those spatially aligned with eye blinks or movements. A maximum of five components were excluded from each dataset, a measure taken to preserve the neural signal’s authenticity. Artifact removal was further refined using the rejection extremum method, eliminating any EEG components that fell outside the ± 100 µV range. Following this meticulous curation, data were recomputed to conform to a whole-brain average reference. Before advancing to microstate analysis, a manual inspection was performed to validate the data's quality, ensuring its reliability for subsequent study phases [29].

### Microstate analysis

Microstate analysis was performed using the EEGLAB microstates 3.0 plugin (https://www.biorxiv.org/content/10.1101/289850v1). Following the application of the average reference, the instantaneous peak values of Global Field Power were extracted from the bandpass-filtered EEG data (2–20 Hz) [10]. The EEG time points corresponding to these peaks were then input into an atomized and agglomerated hierarchical clustering (AAHC) algorithm, resulting in the acquisition of the average microstate topology for each category [30]. The optimal number of microstates (*k* = 4) was determined by a multi-criteria approach, which included not only the consideration of Global Explained Variance (GEV) but also the application of a stopping criterion for GEV's rate of increase, model complexity, interpretability, comparison with existing literature, and a specific GEV threshold for minimal incremental gain. This comprehensive methodology ensures that the microstates effectively represent EEG data while maintaining model parsimony and interpretability [31, 32]. During clustering, we accounted for polarity by treating inverse microstates equivalently. The topographies for each microstate category were averaged across participants to create a set of template maps. Individual EEG datasets were backfitted against these templates by assigning each EEG data point to the template with the highest spatial correlation. This method ensured that individual variations within the EEG data were maintained while still allowing for group-level analysis.

To define the microstate parameters, we measured the duration (milliseconds of persistence), occurrence (frequency per second), coverage (time proportion represented), and transition probabilities (likelihood of state changes). These metrics were calculated to assess the dynamics and prevalence of each microstate within the EEG recordings.

### Statistical analysis

SPSS 25 (IBM Corp., Armonk, NY, USA) was used for statistical analysis. The chi-square test was used to compare sex differences between groups. Other normal and non-normal measurements were compared between groups using an independent sample *t*-test and the Mann–Whitney *U* test, respectively. Concurrently, we applied the False Discovery Rate (FDR) method to calibrate various types of micro-state indicators through comparative analysis at the group level. Correlation analysis was performed using partial correlation analysis. Sex, age, and years of education were used as covariates to analyze intergroup differences in microstate parameters (duration, occurrence, coverage, and transition probability) in relation to neuropsychological assessments and cerebrospinal fluid biomarkers (Aβ_1-42_, Aβ_1-40_, phosphorylated-tau, total-tau, and neurofilament light chain protein) in AD and HC. Stepwise multiple linear regression was applied to validate the results of the partial correlation analysis and explore whether microstate pattern impairment could predict CSF biomarkers in patients with AD.

In our study, we have refined our approach to evaluating EEG microstate features for the diagnosis and classification of patients with AD. Initially, these features were fed into a binary logistic regression model. However, to ensure robustness and address potential overfitting issues, we have now employed a decision tree model. The data was first divided into training and testing sets in an 8:2 ratio through a random split, ensuring that both sets are representative of the overall dataset. For the training set, we applied a five-fold cross-validation method. This approach allowed us to evaluate the model's performance on different subsets of the training data, enhancing the reliability of our findings. Although we did not perform hyperparameter tuning in this study, aiming to assess the baseline performance of the model, the derived characteristic values from this refined method were then used to construct Receiver Operating Characteristic (ROC) curves, facilitating a more rigorous assessment of the model's diagnostic and classification capabilities in distinguishing AD.

## Results

### Clinical and demographic data

Table 1 and Supplementary Table 1 provide a comprehensive overview of the demographics, neuropsychological assessment scores, and AD biomarkers for patients with AD and HC. There were no significant differences in sex(25 male/ 31 female vs. 14 male/ 24 female, χ^2^ = 0.567, *P* = 0.451), age(62.22 ± 8.41 vs. 60.28 ± 7.10, Z = -0.183, *P* = 0.855), HAMA, or HAMD between the AD and HC group (*P* > 0.05). However, the AD group exhibited impaired overall cognitive function and multi-domain cognitive function compared to the HC group (all *P* < 0.05).

**Table 1:** Demographic and clinical data of AD and HC(‾x ± s)

	AD (*n* = 56)	HC (*n* = 38)	χ^2^/ T/Z Value	*P* Value
Demographic characteristics				
Gender (male/female) ^a^	25/31	14/24	0.567	0.451
Age (years) ^c^	62.22 ± 8.409	60.28 ± 7.096	-0.183	0.855
Education (years) ^b^	8.23 ± 4.355	12.41 ± 2.649	-5.370	*P* < 0.001
Clinical symptom measures				
Mini-Mental State Examination ^c^	18.72 ± 6.364	28.46 ± 1.401	-6.547	*P* < 0.001
Montreal Cognitive Assessment ^b^	13.39 ± 6.435	26.39 ± 1.833	-13.809	*P* < 0.001
Lawton-Brody Activities of Daily Living ^c^	28.31 ± 7.315	20.18 ± 0.772	-6.757	*P* < 0.001
Clinical Dementia Rating ^c^	1.00 ± 0.454	0.09 ± 0.195	-7.352	*P* < 0.001
Global Deterioration Scale ^c^	3.65 ± 0.590	1.79 ± 0.568	-7.431	*P* < 0.001
Hamilton Anxiety Rating Scale ^c^	3.12 ± 4.736	3.67 ± 3.563	-1.471	0.141
Hamilton Depression Rating Scale ^c^	2.73 ± 3.578	2.44 ± 3.117	-0.071	0.944
Cerebrospinal fluid examination (pg/ml)				
Aβ (1–42)	729.61 ± 395.492	-	-	-
Aβ (1–40)	8885.68 ± 3651.200	-	-	-
Aβ (1–42) /Aβ (1–40)	0.09 ± 0.043	-	-	-
P-Tau181	111.94 ± 51.626	-	-	-
T-Tau	546.01 ± 262.743	-	-	-
NF-Light	1408.62 ± 1097.065			

^a^ χ^2^ test; ^b^ Independent sample t test; ^c^ Mann–Whitney U test

*AD* = Alzheimer's disease; *HC* = Healthy controls; *P-Tau181* = Phosphorylated tau-181 protein; *T-Tau* = Total tau protein; *NF-Light* = Neurofilament light chain protein

### EEG microstate

**Fig. 1:**
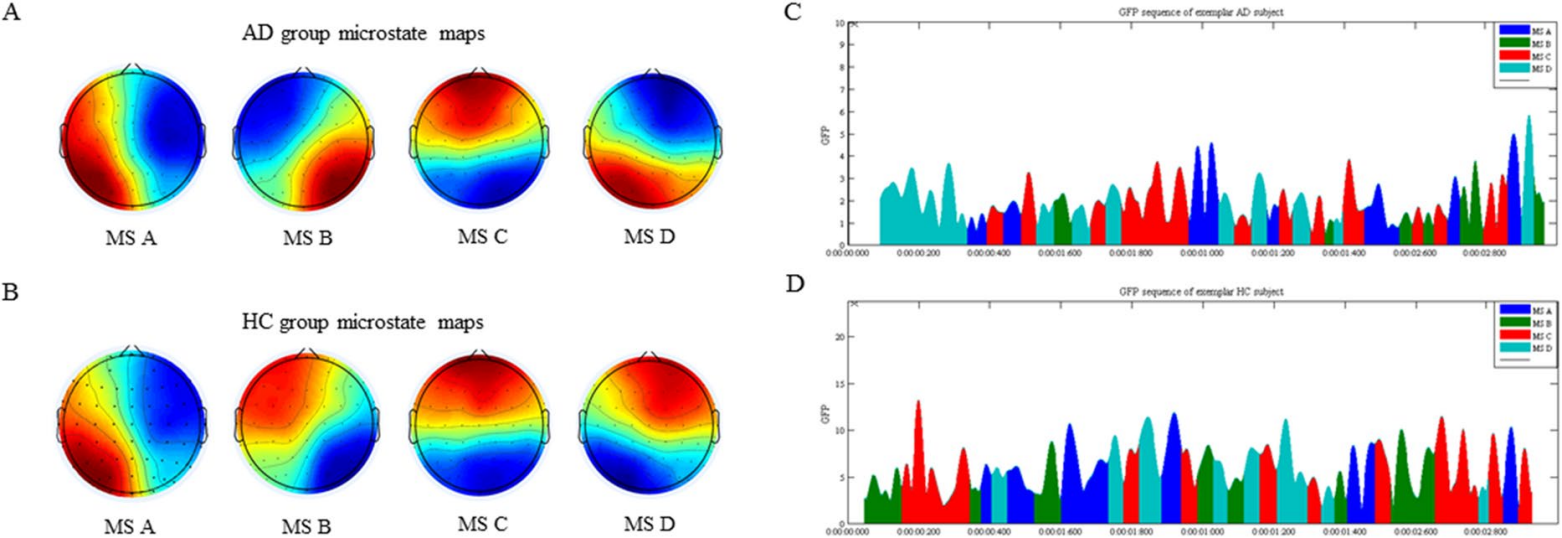
(**A**) AD group microstate maps and (**B**) HC group microstate maps. EEG signals were clustered into four subject-specific terrains, and their time series were calculated. Topographic and temporal features were extracted from microstate activity. Example of Global Electric Field Power time course within a 2-s interval of continuous EEG signal from (**C**) a 56-year-old male patient with AD and (**D**) a 59-year-old male HC. Abbreviations: AD = Alzheimer's disease; HC = Healthy controls; GFP = Global Field Power; MS = microstate

Table 2 provides an overview of EEG microstate patterns in the AD and HC groups. The median number of optimal microstate categories in patients with AD and HC was 4. Therefore, we set the number of microstate categories to four, labeled A, B, C, and D [10]. The mean GEV of the four microstates in each group was 0.774 ± 0.048 in the AD group and 0.785 ± 0.047 in the HC group (Fig. 1).

**Table 2:** EEG microstate data in AD and HC groups(‾x ± s)

	AD (*n* = 56)	HC (*n* = 38)	*T/Z* Value	*P* Value	*P*_FDR_corr_ Value
Global explained variance ^b^	0.774 ± 0.048	0.785 ± 0.047	-0.140	0.899	0.899
Total time ^c^	271.101 ± 67.674	264.591 ± 56.180	-0.146	0.884	1.000
Duration (s)					
Duration A ^c^	0.068 ± 0.016	0.069 ± 0.042	-1.656	0.098	0.123
Duration B ^c^	0.067 ± 0.015	0.063 ± 0.009	-1.387	0.166	0.166
Duration C ^c^	0.069 ± 0.015	0.063 ± 0.016	-2.412	0.016	0.040*
Duration D ^c^	0.068 ± 0.016	0.061 ± 0.015	-2.465	0.014	0.070
Mean Duration ^c^	0.071 ± 0.011	0.068 ± 0.017	-2.057	0.040	0.067
Occurrence (/s)					
Occurrence A ^b^	3.720 ± 0.822	4.026 ± 1.010	-1.611	0.111	0.185
Occurrence B ^b^	3.805 ± 0.864	4.262 ± 0.894	-2.481	0.015	0.075
Occurrence C ^c^	3.639 ± 0.887	3.872 ± 0.841	-1.456	0.145	0.181
Occurrence D ^b^	3.745 ± 0.827	3.620 ± 0.874	0.704	0.483	0.483
Mean Occurrence ^c^	14.909 ± 1.925	15.779 ± 1.96	-2.281	0.023	0.145
Contribution					
Contribution A ^c^	0.252 ± 0.092	0.266 ± 0.127	-0.562	0.574	0.765
Contribution B ^b^	0.251 ± 0.080	0.265 ± 0.075	-0.862	0.391	0.782
Contribution C ^b^	0.245 ± 0.079	0.245 ± 0.091	0.027	0.978	0.978
Contribution D ^c^	0.251 ± 0.089	0.224 ± 0.084	-1.502	0.133	0.532
Transition probability					
TP of A–-B ^c^	0.088 ± 0.039	0.101 ± 0.045	-1.479	0.139	0.556
TP of A–-C ^b^	0.067 ± 0.010	0.072 ± 0.022	-1.082	0.285	0.570
TP of A–-D ^c^	0.084 ± 0.029	0.073 ± 0.026	-1.564	0.118	0.708
TP of B–-A ^c^	0.089 ± 0.037	0.099 ± 0.045	-0.886	0.376	0.546
TP of B–-C ^b^	0.081 ± 0.035	0.087 ± 0.034	-0.796	0.428	0.514
TP of B–-D ^b^	0.074 ± 0.011	0.070 ± 0.021	0.958	0.342	0.586
TP of C–-A ^c^	0.068 ± 0.010	0.074 ± 0.021	-1.880	0.060	0.720
TP of C–-B ^c^	0.082 ± 0.035	0.086 ± 0.034	-0.539	0.590	0.590
TP of C–-D ^b^	0.084 ± 0.039	0.077 ± 0.038	0.830	0.409	0.545
TP of D–-A ^c^	0.082 ± 0.029	0.073 ± 0.025	-1.410	0.159	0.477
TP of D–-B ^b^	0.074 ± 0.011	0.070 ± 0.020	1.198	0.236	0.566
TP of D–-C ^c^	0.086 ± 0.039	0.079 ± 0.038	-0.655	0.513	0.560

^b^ Independent sample t test; ^c^ Mann–Whitney U test; * After FDR correction, the difference was still statistically significant

*AD* = Alzheimer's disease; *HC* = Healthy controls; *TP* = Transition probability; *FDR* = False Discovery Rate

**Fig. 2:**
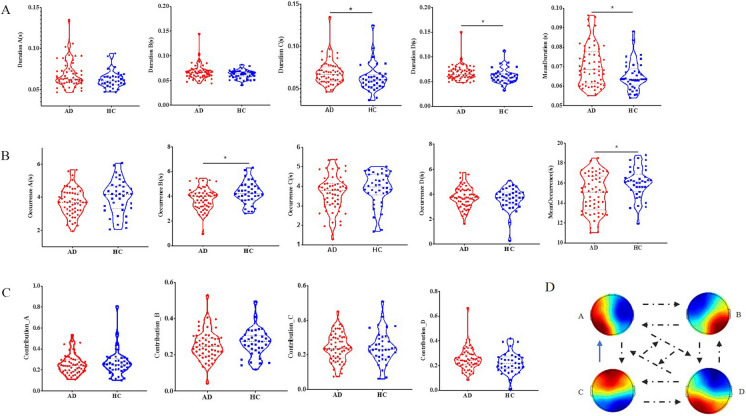
Microstate metrics. (**A**) Difference in the duration of each pair of microstates and mean duration between the two groups, measured in milliseconds, representing how long a given microstate type persists. (**B**) Intergroup differences in the occurrence of each pair of microstates and mean occurrence, quantifying how frequently a specific microstate type repeats per second. (**C**) Intergroup differences in the coverage of each pair of microstates, indicating the proportion of time each microstate type covers. (**D**) Differences in TP among groups, referring to the probability of mutual transition between microstates. The black dotted arrows connect the microscopic states of TP with no significant difference. The blue arrow indicates that pairwise TP C → A has a decreasing trend (*P* = 0.60). Abbreviations: TP = Transition probability; Note: Before FDR correction, * *P*-value significant at < 0.05; ** *P*-value significant at < 0.01

Compared with HC, the duration of microstates C (z = -2.412, *P* = 0.016) and microstates D (z = -2.465, *P* = 0.014) and mean duration (z = -2.057, *P* = 0.040) in patients with AD were significantly longer. Additionally, the occurrence of microstates B (t = -2.481, *P* = 0.015) and the mean occurrence (z = -2.281, *P* = 0.023) were considerably reduced. Furthermore, there was a reduction in the transition probability from microstates C to A (z = -1.880, *P* = 0.060). After FDR correction, only the duration of microstate C was still statistically significant (*P* = 0.040), while the duration of microstate D (*P* = 0.070), mean duration (*P* = 0.067), and the occurrence of microstate B (*P* = 0.075) only showed a difference trend. Notably, the duration, occurrence, contribution, and transition probability of the other microstate types did not show significant abnormalities (all *P* > 0.05) (Fig. 2).

### Relation between EEG microstate and clinical/cognitive function

Table 3 and Supplementary Table 2 present the correlation analysis of different microstate patterns with cognitive function and clinical characteristics in the AD and HC groups, respectively. After adjusting for sex, age, and years of education, the duration of microstates C in the AD group was significantly positively correlated with SCWT-D (*ρ* = 0.448, *P* = 0.010) and SCWT-W (*ρ* = 0.356, *P* = 0.045). The mean duration was significantly positively correlated with ADL (*ρ* = 0.349, *P* = 0.050), SCWT-D (*ρ* = 0.393, *P* = 0.026), SCWT-W (*ρ* = 0.479, *P* = 0.006) and SCWT-CW (*ρ* = 0.366, *P* = 0.040). The mean occurrence was significantly negatively correlated with SCWT-W (*ρ* = -0.385, *P* = 0.030). The transition probability of microstates C to A was significantly negatively correlated with VFT-S (*ρ* = -0.402, *P* = 0.023). There were no significant correlations between the other abnormal microstate patterns, clinical symptoms, and cognitive function (all *P* > 0.05).

**Table 3:** Relationship between EEG microstate abnormalities and cognitive function in AD group

		Duration_C	Duration_D	Mean Duration	Occurrence_B	Mean Occurrence	TP of C–-A
Clinical symptom measures							
Mini-Mental State Examination	*ρ* Value	-0.255	0.150	-0.289	-0.213	0.251	-0.330
	*P* Value	0.160	0.411	0.109	0.242	0.166	0.065
Montreal Cognitive Assessment	*ρ* Value	-0.237	0.247	-0.286	-0.256	0.238	-0.308
	*P* Value	0.192	0.172	0.113	0.157	0.189	0.086
Lawton-Brody Activities of Daily Living	*ρ* Value	0.213	0.035	0.349	-0.018	-0.307	0.149
	*P* Value	0.243	0.848	0.050*	0.923	0.087	0.417
Clinical Dementia Rating	*ρ* Value	0.149	0.000	0.297	0.107	-0.277	0.038
	*P* Value	0.415	0.998	0.099	0.561	0.125	0.838
Global Deterioration Scale	*ρ* Value	0.254	-0.111	0.292	0.172	-0.263	0.165
	*P* Value	0.161	0.544	0.105	0.346	0.146	0.366
Multi-domain Cognition Assessments							
Memory function Assessment							
CAVLT-Immediate	*ρ* Value	-0.184	0.087	-0.231	-0.118	0.181	-0.196
	*P* Value	0.313	0.638	0.204	0.522	0.322	0.282
CAVLT-Delay	*ρ* Value	-0.166	0.000	-0.280	-0.107	0.262	-0.092
	*P* Value	0.363	0.998	0.121	0.559	0.148	0.616
CAVLT-Recognition	*ρ* Value	-0.004	-0.097	-0.192	-0.077	0.149	0.018
	*P* Value	0.982	0.598	0.293	0.677	0.415	0.920
Attention function Assessment							
Digital Span Test -Forward	*ρ* Value	-0.113	0.148	-0.245	-0.076	0.207	-0.162
	*P* Value	0.540	0.419	0.177	0.678	0.256	0.374
Digital Span Test -Backward	*ρ* Value	-0.250	0.048	-0.260	-0.065	0.244	-0.005
	*P* Value	0.167	0.795	0.151	0.723	0.178	0.978
Executive function Assessment							
SCWT- Dot	*ρ* Value	0.448	-0.196	0.393	0.120	-0.302	0.288
	*P* Value	0.010*	0.283	0.026*	0.513	0.093	0.110
SCWT-Word	*ρ* Value	0.356	-0.219	0.479	0.144	-0.385	0.229
	*P* Value	0.045*	0.229	0.006**	0.431	0.030*	0.207
SCWT- Color Word	*ρ* Value	0.225	-0.241	0.366	0.239	-0.334	0.197
	*P* Value	0.216	0.184	0.040*	0.188	0.062	0.280
Language function Assessment							
Verbal Fluency Test -Letter	*ρ* Value	-0.098	0.024	-0.289	0.063	0.282	-0.131
	*P* Value	0.594	0.895	0.108	0.730	0.118	0.476
Verbal Fluency Test-Sematic	*ρ* Value	-0.159	0.136	0.010	-0.103	-0.067	-0.402
	*P* Value	0.384	0.459	0.955	0.575	0.718	0.023*

Adjusted for gender, age, and years of education; * *P* value significant at < 0.05 ** P value significant at < 0.01

*CAVLT* = Chinese version of the Auditory Verbal Learning Test; *SCWT* = Stroop Color Word Test; *TP of C–-A* = Transition probability of microstate C to A

After adjusting for sex, age, and years of education, the duration of microstates C in the HC group was significantly positively correlated with MMSE (*ρ* = 0.634, *P* = 0.015) and MoCA (*ρ* = 0.525, *P* = 0.037). The transition probability of microstates C to A was negatively correlated with SCWT-C (*ρ* = -0.887, *P* < 0.001) and SCWT-CW (*ρ* = -0.731, *P* = 0.003). Other microstate patterns were not significantly associated with cognitive function (all *P* > 0.05).

### Potential predictors of CSF biomarkers and disease classification in AD

Table 4 shows the correlation analysis between the differences in microstate patterns and CSF biomarkers in the AD group. After adjusting for sex, age, and years of education, the duration of microstates C in the AD group was negatively correlated with Aβ_1-42_(*ρ* = -0.387, *P* = 0.035) and Aβ_1-40_ (*ρ* = -0.362, *P* = 0.050). The mean duration was negatively correlated with Aβ_1-42_(*ρ* = -0.401, *P* = 0.028). The mean occurrence was positively correlated with Aβ_1-42_ (*ρ* = -0.453, *P* = 0.012).

**Table 4:** Relationship between EEG microstate abnormalities and CSF in AD group

		Duration_C	Duration_D	Mean Duration	Occurrence_B	Mean Occurrence	TP of C–-A
Cerebrospinal fluid Biomarker							
Aβ (1_42)	*ρ* Value	-0.387	-0.053	-0.401	0.017	0.453	0.046
	*P* Value	0.035*	0.779	0.028*	0.927	0.012*	0.811
Aβ (1_40)	*ρ* Value	-0.362	-0.278	-0.160	0.311	0.183	0.157
	*P* Value	0.050*	0.137	0.398	0.094	0.333	0.406
Aβ (1_42) / Aβ (1_40)	*ρ* Value	-0.110	0.126	-0.274	-0.197	0.297	-0.051
	*P* Value	0.563	0.508	0.143	0.297	0.111	0.789
P_Tau181	*ρ* Value	0.077	-0.097	0.126	0.147	-0.173	0.171
	*P* Value	0.685	0.610	0.506	0.437	0.361	0.366
T_Tau	*ρ* Value	-0.069	-0.264	-0.060	0.272	0.040	0.287
	*P* Value	0.719	0.159	0.751	0.145	0.832	0.123
NF-Light	*ρ* Value	0.263	0.015	0.137	-0.096	-0.123	-0.234
	*P* Value	0.161	0.936	0.470	0.614	0.519	0.213

Adjusted for gender, age, and years of education; * *P* value significant at < 0.05 ** *P* value significant at < 0.01

*TP of C–-A* = Transition probability of microstate C to A; *P_Tau181* = Phosphorylated-tau181; *Aβ* = amyloid beta; *T_Tau* = Total-tau; *NF-Light* = Neurofilament light chain protein

**Fig. 3:**
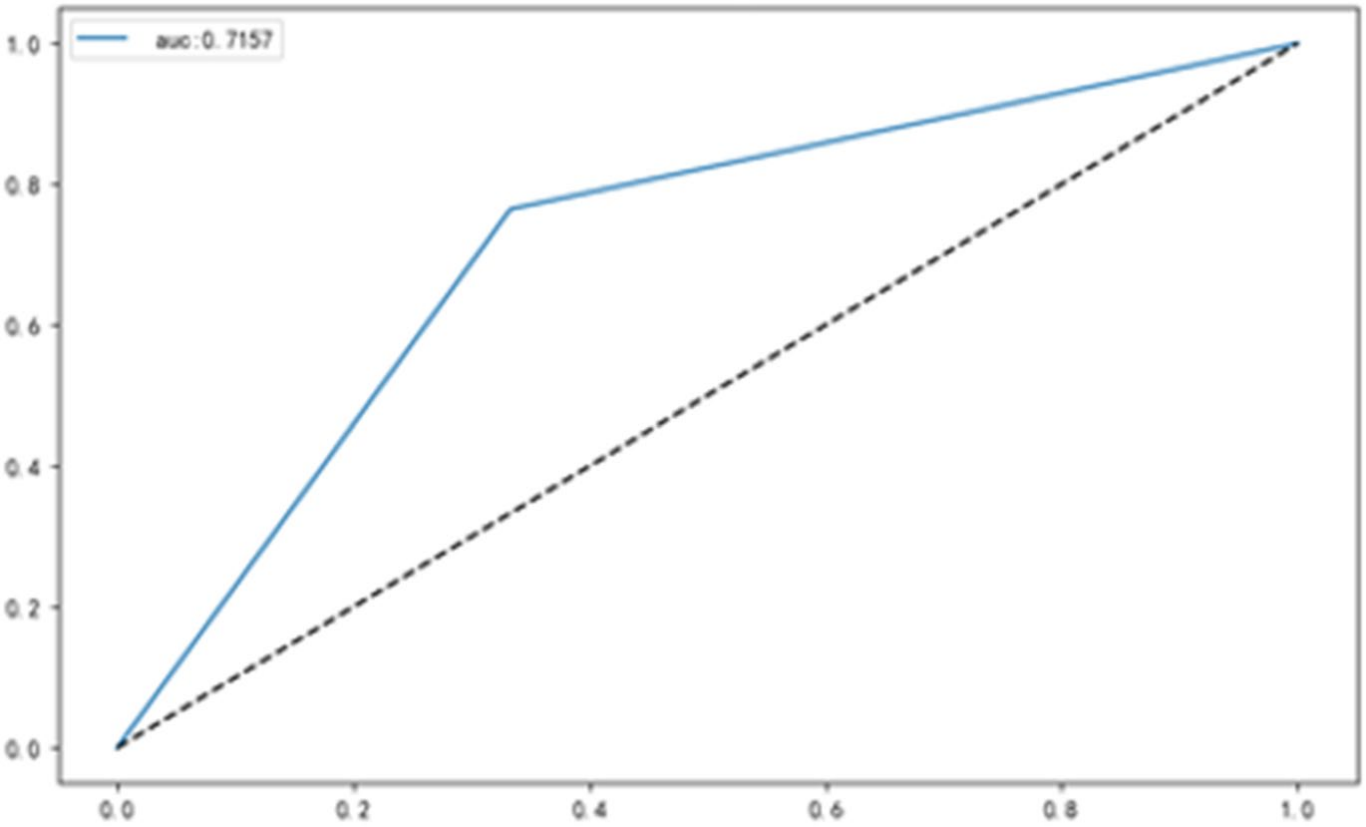
Classification results based on the microstate parameter. We used EEG microstates for predictive classification of AD, and the results showed that the accuracy, specificity, sensitivity and AUC of prediction were 72%, 67%, 76%, and 72%, respectively. Abbreviations: AD = Alzheimer's disease; EEG = Electroencephalogram

In addition, we included six different EEG microstate indicators, sex, age, and years of education, as independent variables in the multiple regression model for stepwise regression. Supplementary Table 3a shows that the mean duration (Beta = 2.497, 95% Confidence interval: 179.857 ~ 794.936, *t* = 3.241, *P* = 0.003), occurrence of microstates B (Beta = -0.477, 95% Confidence interval: -343.679 ~ -54.225, *t* = -2.812, *P* = 0.009) and mean occurrence (Beta = 1.908, 95% Confidence interval: 13,015.144 ~ 119,827.546, *t* = 2.544, *P* = 0.017) were significant predictors of CSF Aβ_1-42_ in AD. Supplementary Table 3b shows that microstates C's duration (Beta = -0.380, 95% Confidence interval: -218618.220 ~ -21786.342, *t* = -2.491, *P* = 0.018) significantly predicted CSF Aβ_1-40_ in AD. Finally, we used all the EEG microstate values to predict AD. In contrast, the decision tree model, which was also subjected to five-fold cross-validation, yielded an accuracy of 72%, specificity of 67%, and sensitivity of 76% (Table 5) (Fig. 3).

**Table 5:** Performance indicators of the model on the test set

	Precision	Recall	F1-score	Support
HC	0.67	0.67	0.67	12
AD	0.76	0.76	0.76	17
Accuracy			0.72	29
Macro avg	0.72	0.72	0.72	29
Weighted avg	0.72	0.72	0.72	29

*AD* = Alzheimer's disease; *HC* = Healthy controls

## Discussion

In this study, we used EEG microstates to assess the temporal characteristics of brain activity on subsecond timescales. We investigated changes in brain dynamics among patients with AD and examined the relationships between EEG microstate dynamics, cognitive function, and CSF biomarkers. Our findings indicated that the duration of microstates in patients with AD was prolonged, microstate occurrence decreased, and disturbance in microstates were associated with executive function and the severity of dementia in AD, as well as with the concentration of Aβ in CSF. Furthermore, the integration of all microstate measures for predictive classification demonstrated moderate effectiveness in distinguishing patients with AD from control subjects. Our study proposed for the first time that the disturbance of resting EEG microstate dynamics in patients with AD could serve as an indicator for predicting the degree of Aβ deposition and for disease diagnosis, making it an effective complementary tool for AD biomarkers.

Our study shows that microstate dynamics were significantly slowed in patients with AD, suggesting that a low-dynamics brain may be a feature of AD. Compared with HC, patients with AD exhibited prolonged periods within the same microstate, indicating a slower transition between states. In patients with AD, the duration of microstates C and D, as well as the mean duration, were prolonged, while the occurrence of microstate B and the mean occurrence decreased. Additionally, the probability of transition from microstate C to A decreased. Previous studies on EEG microstates in patients with AD yielded inconsistent findings; some reported decreased microstate durations [27], while others found no statistical differences in microstate patterns between patients with AD and HC [33, 34]. Recent studies have confirmed that microstate dynamics slow in patients with AD, but the changes in microstate patterns are not entirely consistent [12, 20, 35]. These discrepancies may be attributed to variations in sample size and EEG microstate analysis methods.

Additionally, we observed a decreasing trend in the probability of transitioning from microstate C to A in patients with AD. Some studies have suggested that, during normal brain development, the coverage of asymmetric microstates (classes A and B) decreases, while that of symmetrical microstates (classes C and D) increases. However, the microstates change at various stages of cognitive impairment [20, 21]. Our findings can be interpreted as a suppressed transition from symmetry to asymmetry in patients compared to healthy controls, which differs from previous research [12, 20, 25]. We speculate that this change in transition probability could reflect compensatory brain activity in these patients. However, this change was not statistically significant, and further expansion of the sample size is necessary to verify the results. Furthermore, our study also found that combining all microstate indicators resulted in moderate accuracy in disease classification, which further confirmed that microstate disruption in patients with AD represents a characteristic change and can serve as a supplementary basis for disease diagnosis and classification.

We further performed a correlation analysis and identified a close relationship between changes in microstate patterns and cognitive function. Previous studies have shown that EEG microstates can be used to characterize and understand neuronal activity in specific brain networks [36]. In addition, several studies have demonstrated spatial correlations between the four typical microstate categories and fMRI resting state networks, which also have functional implications [16, 36, 37]. Previous fMRI investigations in patients with AD have reported impaired functional connectivity within the resting-state brain network, including both increased and decreased functional connections [6, 8]. The aberrant organization and function of the three core neurocognitive networks—the default mode network, central executive network, and salience network—have been recognized as prominent features of AD [38]. Some studies have indicated that microstates A and B are associated with the auditory and visual networks [16], microstate C belongs to both the salience network and the subnetwork of the default mode network [37], and microstate D may overlap with the executive control network [10, 25, 37]. Therefore, we hypothesized that altered EEG microstate patterns may be a neuroelectrophysiological mechanism underlying impaired cognitive function, which was supported by our study findings.

We observed that, as the duration of microstate C increased, the executive function of patients with AD worsened. Similarly, with a longer mean duration, there was a decrease in mean occurrence, leading to poorer executive function and more severe clinical symptoms. Recent reviews have suggested that microstate C is associated with activity in the cognitive control network, mainly the salience network, and involves activation of the anterior cingulate gyrus, which is part of the executive control network, as well as the insula. Simultaneously, microstate C is believed to reflect a component of the default mode network, often referred to as the task-negative network, which exhibits reduced activity during cognitive task performance [10, 36]. Previous studies have identified decreased functional connectivity of the left anterior cingulate region in patients with AD, explaining the correlation between microstate C and executive function [6]. Mean duration and occurrence serve as indicators of the stability of underlying neural components [19]. When these indicators deviate from the normal, the functional connectivity of the brain network corresponding to the disturbance of the four microstate patterns is impaired, affecting the cognitive function of patients with AD. Furthermore, we observed that the transition probability of microstate C to A correlated with language function. The transition probability reflects the dynamic changes and information exchange within the brain’s neural network, indicating the frequency of information flow between brain networks. This may be related to resource allocation and switching in the brain during complex tasks [19, 21]. When the network structure is compromised, connections between brain network functions are disrupted, impacting the brain's behavioral function [39].

We analyzed the relationship between EEG microstates and CSF biomarkers and found that the duration of microstate C, mean duration, and mean occurrence were related to the pathology of AD, but the correlation was only low to moderate. In our refined regression model, we incorporated microstate indicators showing different trends and discovered that the occurrence of microstate B and the duration of microstate C, along with their mean duration and mean occurrence, were predictive of Aβ concentration in the CSF of patients with AD. Although these microstates did not show significant associations individually, our comprehensive model revealed a statistically significant correlation with the CSF biomarker when considering these predictors collectively. This finding highlights the necessity of adopting a multifaceted approach to understanding the complex interrelationships in biological data. These microstate characteristics could serve as EEG markers reflecting changes in CSF pathology, specifically concerning Aβ concentration. Previous research has established a connection between decreased Aβ levels in CSF and increased amyloid accumulation in the brain [40]. The amyloid cascade hypothesis, central to AD pathogenesis, suggests a causal relationship between amyloid accumulation and synaptic dysfunction within the Alzheimer's continuum [41]. Furthermore, disruptions in the large-scale networks of functional brain tissues have been associated with AD progression [42]. Therefore, we propose that the large-scale disruption of brain networks due to AD pathology is related to the disturbance of microstate patterns, providing a rationale for utilizing duration and mean occurrence as predictors of CSF Aβ levels. In addition, a previous study indicated that pathological changes in AD predominantly affect the default mode network region [4], while another study suggested that AD pathology may initially manifest in the higher-order visual association region, with the visual network serving as a sensitive marker of AD progression [43, 44]. This may explain the observed correlation between CSF Aβ levels and microstates C/B. The intricate collaboration of large-scale functional brain networks in task performance can lead to cognitive impairment when connectivity is compromised [4–6]. Therefore, we propose that pathological changes in AD lead to a wide range of functional brain network disorders, affecting EEG microstate patterns and contributing to cognitive decline. However, to substantiate these claims, further investigations are warranted, including mediation effect analyses with expanded sample sizes, as well as CSF and fMRI-EEG studies.

Our study has some limitations. First, we did not collect CSF samples from the HC group; therefore, we were unable to conduct a comprehensive two-factor verification of the interaction between clinical factors and biomarker classification in determining EEG microstates. Second, we did not include patients with early or preclinical AD; therefore, we could not explore whether the identified microstate features could consistently describe decline at all stages of AD. Finally, some patients with AD were taking acetylcholinesterase inhibitors, which may have affected the EEG data [45]. The comparison between the AD and control groups may have been influenced by the use of these drugs. Further prospective studies on medication use are required to address this issue.

In summary, we observed that the duration and occurrence of microstate patterns were disrupted in patients with AD compared to those in HC. These disruptions were associated with changes in executive and daily living abilities. Additionally, our study is the first to suggest that alterations in microstate patterns can potentially serve as predictors of Aβ concentration in CSF, making EEG microstate markers valuable non-invasive tools for AD diagnosis. In the future, large-scale cohort and longitudinal studies should be conducted during the early or preclinical stages of AD to investigate whether changes in EEG microstate indicators in patients with AD can assist in CSF examination and predict the onset of MCI or AD.

### Supplementary Information

Below is the link to the electronic supplementary material.

Supplementary file1 (DOCX 33 KB)

## Data Availability

All datasets generated for this study are included in the manuscript and/or the supplementary files.
