# Supplementary material for: Abnormal EEG microstates in Alzheimer’s disease: predictors of β-amyloid deposition degree and disease classification
Source: GeroScience. 2024 May 10;46(5):4779–92. doi: 10.1007/s11357-024-01181-5 (PMC11336126; doi:10.1007/s11357-024-01181-5)
Supplement: Supplementary file 1 — Supplementary file1 (DOCX 33 KB) [file 11357_2024_1181_MOESM1_ESM.docx]

| **Supplementary Table 1. Neuropsychological tests of AD and HCs（‾x±s）** | | | | |
| --- | --- | --- | --- | --- |
|  | **AD（n=56）** | **HC（n=37）** | ***χ^2^/ T/Z* Value** | ***P* Value** |
| **Multi-domain Cognition Assessments** | | | | |
| **Memory function Assessment** | | | | |
| CAVLT- Immediate ^b^ | 3.70±2.239 | 9.00±1.827 | ***-10.415*** | ***P<0.001*** |
| CAVLT- Delay ^c^ | 1.46±2.639 | 9.85±2.824 | ***-6.906*** | ***P<0.001*** |
| CAVLT- Recognition ^c^ | 11.51±3.806 | 14.33±1.038 | ***-4.512*** | ***P<0.001*** |
| **Attention function Assessment** | | | | |
| Digital Span Test -Forward ^b^ | 6.13±1.596 | 7.74±1.289 | ***-4.475*** | ***P<0.001*** |
| Digital Span Test-Backward ^c^ | 3.13±1.244 | 5.19±0.834 | ***-5.922*** | ***P<0.001*** |
| **Executive function Assessment** | | | | |
| SCWT-Dot(s) ^b^ | 38.18±27.022 | 15.11±2.178 | ***5.436*** | ***P<0.001*** |
| SCWT -Word(s) ^c^ | 55.65±52.490 | 18.12±3.451 | ***-5.450*** | ***P<0.001*** |
| SCWT-Color Word(s) ^c^ | 72.32±45.754 | 30.55±10.408 | ***-4.812*** | ***P<0.001*** |
| **Language function Assessment** | | | | |
| Verbal Fluency Test -Letter ^b^ | 3.84±2.946 | 7.58±3.220 | ***-4.038*** | ***P<0.001*** |
| Verbal Fluency Test-Sematic ^c^ | 10.04±4.325 | 19.93±4.649 | ***-5.356*** | ***P<0.001*** |
| **Note:** ^a^ χ^2^ test; ^b^ Independent sample t test; ^c^ Mann–Whitney U test  **Abbreviations:** AD = Alzheimer's disease; CAVLT: Chinese version of the Auditory Verbal Learning Test; SCWT: Stroop Color Word Test. | | | | |

| **Supplementary Table 2. Relationship between EEG microstate and cognitive function in HC group** | | | | | | | |
| --- | --- | --- | --- | --- | --- | --- | --- |
|  |  | **Duration_C** | **Duration_D** | **MeanDuration** | **Occurrence_B** | **MeanOccurrence** | **TP of C---A** |
| Mini-Mental State Examination | *ρ* Value | ***0.634*** | 0.145 | 0.419 | -0.386 | -0.424 | 0.323 |
|  | *P* Value | ***0.015**** | 0.620 | 0.136 | 0.173 | 0.130 | 0.261 |
| Montreal Cognitive Assessment | *ρ* Value | 0.469 | 0.197 | 0.465 | -0.192 | -0.489 | 0.082 |
|  | *P* Value | 0.091 | 0.499 | 0.094 | 0.511 | 0.076 | 0.781 |
| **Multi-domain Cognition Assessments** | | | | | | | |
| **Memory function Assessment** | | | | | | | |
| CAVLT-Immediate | *ρ* Value | 0.402 | -0.113 | 0.057 | -0.235 | -0.124 | 0.511 |
|  | *P* Value | 0.154 | 0.701 | 0.847 | 0.418 | 0.672 | 0.062 |
| CAVLT-Delay | *ρ* Value | 0.207 | -0.242 | 0.188 | -0.120 | -0.260 | 0.526 |
|  | *P* Value | 0.477 | 0.404 | 0.520 | 0.682 | 0.370 | 0.053 |
| CAVLT-Recognition | *ρ* Value | -0.141 | -0.028 | 0.369 | -0.044 | -0.397 | 0.083 |
|  | *P* Value | 0.632 | 0.924 | 0.194 | 0.880 | 0.160 | 0.779 |
| **Attention function Assessment** | | | | | | | |
| Digital Span Test -Forward | *ρ* Value | -0.367 | -0.103 | -0.098 | 0.220 | 0.110 | -0.241 |
|  | *P* Value | 0.197 | 0.727 | 0.738 | 0.450 | 0.708 | 0.406 |
| Digital Span Test -Backward | *ρ* Value | -0.148 | -0.060 | 0.324 | -0.045 | -0.329 | -0.007 |
|  | *P* Value | 0.614 | 0.839 | 0.258 | 0.878 | 0.251 | 0.981 |
| **Executive function Assessment** | | | | | | | |
| SCWT- Dot | *ρ* Value | -0.165 | -0.050 | -0.184 | 0.129 | 0.150 | -0.161 |
|  | *P* Value | 0.573 | 0.864 | 0.528 | 0.660 | 0.610 | 0.582 |
| SCWT-Word | *ρ* Value | -0.235 | 0.481 | 0.252 | -0.007 | -0.127 | ***-0.887*** |
|  | *P* Value | 0.419 | 0.082 | 0.386 | 0.980 | 0.666 | ***<0.001***** |
| SCWT- Color Word | *ρ* Value | -0.032 | 0.418 | 0.277 | -0.002 | -0.177 | ***-0.731*** |
|  | *P* Value | 0.914 | 0.137 | 0.338 | 0.994 | 0.546 | ***0.003***** |
| **Language function Assessment** | | | | | | | |
| Verbal Fluency Test -Letter | *ρ* Value | -0.355 | -0.306 | -0.183 | 0.371 | 0.192 | -0.373 |
|  | *P* Value | 0.213 | 0.288 | 0.532 | 0.192 | 0.511 | 0.189 |
| Verbal Fluency Test-Sematic | *ρ* Value | -0.240 | -0.402 | -0.123 | 0.265 | 0.091 | 0.177 |
|  | *P* Value | 0.409 | 0.154 | 0.674 | 0.360 | 0.757 | 0.546 |
| **Note:** Adjusted for gender, age, and years of education; * P value significant at < 0.05 ** P value significant at < 0.01  **Abbreviations:** CAVLT = Chinese version of the Auditory Verbal Learning Test; SCWT = Stroop Color Word Test；TP = Transition probability | | | | | | | |

| **Supplementary Table 3a: The results of multivariate linear regression** | | | | | |
| --- | --- | --- | --- | --- | --- |
|  | **Standard coefficients** | | | **The 95% CI for Beta** | |
|  | **Beta** | **t** | **p** | **Lower** | **Upper** |
| **Predictive Variables adopted in the model** | | | | | |
| Constant |  | -2.670 | 0.012 | -18917.764 | -2506.127 |
| Education (years) | 0.341 | 2.501 | 0.018 | 5.617 | 56.001 |
| Mean Duration | 2.497 | 3.241 | 0.003 | 179.857 | 794.936 |
| Occurrence_B | -0.477 | -2.812 | 0.009 | -343.679 | -54.225 |
| Mean Occurrence | 1.908 | 2.544 | 0.017 | 13015.144 | 119827.546 |
| **Excluded Variables** |  |  |  |  |  |
| Age | 0.168 | 1.175 | 0.250 |  |  |
| Gender (F=1, M=2) | 0.004 | 0.026 | 0.980 |  |  |
| Duration_C | -0.133 | -0.804 | 0.428 |  |  |
| Duration_D | -0.007 | -0.022 | 0.982 |  |  |
| TP of C---A | 0.129 | 0.753 | 0.458 |  |  |
| **Dependent variable:** Aβ (1_42)  **Predictive variables in the model:** Constant, Education (years), Mean Duration, Occurrence_B, Mean Occurrence  **Abbreviations:** TP of C---A = Transition probability of microstate C to A; Aβ = amyloid beta; | | | | | |

| **Supplementary Table 3b: The results of multivariate linear regression** | | | | | |
| --- | --- | --- | --- | --- | --- |
|  | **Standard coefficients** | | | **The 95% CI for Beta** | |
|  | **Beta** | **t** | **p** | **Lower** | **Upper** |
| **Predictive Variables adopted in the model** | | | | | |
| Constant |  | 1.022 | 0.315 | -5776.583 | 17370.620 |
| Age | 0.368 | 2.411 | 0.022 | 29.281 | 350.732 |
| Duration_C | -0.380 | -2.491 | 0.018 | -218618.220 | -21786.342 |
| **Excluded Variables** |  |  |  |  |  |
| Gender (F=1, M=2) | -0.251 | -1.617 | 0.116 |  |  |
| Education (years) | 0.188 | 1.241 | 0.224 |  |  |
| Duration_D | -0.170 | -1.021 | 0.316 |  |  |
| Mean Duration | 0.092 | 0.503 | 0.618 |  |  |
| Occurrence_B | 0.154 | 0.888 | 0.382 |  |  |
| Mean Occurrence | -0.069 | -0.366 | 0.717 |  |  |
| TP of C---A | -0.061 | -0.392 | 0.698 |  |  |
| **Dependent variable:** Aβ (1_40)  **Predictive variables in the model:** Constant, Age, Duration_C  **Abbreviations:** TP of C---A = Transition probability of microstate C to A；Aβ = amyloid beta; | | | | | |
